# Antioxidant Capacity of Anthocyanins and Other Vegetal Pigments: Modern Assisted Extraction Methods and Analysis

**DOI:** 10.3390/antiox11071256

**Published:** 2022-06-26

**Authors:** Noelia Tena, Agustin G. Asuero

**Affiliations:** Departamento de Química Analítica, Facultad de Farmacia, Universidad de Sevilla, Prof. García González 2, 41012 Sevilla, Spain; ntena@us.es

Anthocyanins [1,2], chlorophylls, and carotenoids [3] are pigments responsible for the colour of many fruits, flowers, and plant tissues. In particular, anthocyanins show potential utility as natural dyes and for having a beneficial impact on health (as demonstrated by many epidemiological studies), as in addition to being safe and innocuous molecules, they have antioxidant properties and exert an effect on the gut microbiome. This special issue of Antioxidants contains twelve contributions—eight research articles and four reviews—including recent advances in the field. The interdisciplinary nature of the subject and the breadth of the content presented by the authors make this special issue very interesting and comprehensive. Modern methods of analysis of anthocyanins, their geographical variability, the improvement of their antioxidant properties, the valorisation of by-products, stability studies, and the metabolomics of chlorophylls and carotenoids are the subject of research or review. “Today, science has few boundaries and collaboration is the name of the game” [4].

Urbonaviciene et al. [5] carried out an evaluation of the antioxidant capacity and physicochemical properties of biologically active compounds (total polyphenol and total anthocyanin content) of wild blueberries by developing chemometric tools that make it possible to relate authenticity and quality control to geographical origin. This is the first study of its kind carried out on wild blueberries from several northern European countries.

The production of anthocyanins from blood oranges requires low temperature conditions, thus being a useful postharvest strategy to be applied in hot climates. Peña et al. [6] found a different response in the case of pear and Moorish oranges at the biochemical and molecular level, the changes being more prominent in the latter case. Blood orange has found use in traditional Asian medicine due to the vital bioactivity of the polyphenols it contains.

The red skin colour of some fruits, such as mango and apple, is vital for marketing and consumer acceptance. Alkan et al. [7] sought to determine whether external pre-harvest treatment of apples and mangoes with phenylalanine can promote red skin colouring of the fruit, and experimentally proved the hypothesis to be true, especially when combined with exposure to sunlight. The level of anthocyanin content of the treated peel of Cripps pink or May Kent apples, as determined by HPLC, increased in both cases.

Janulis et al. [8] performed a qualitative and quantitative analysis of anthocyanin and anthocyanidin composition in a variety of cultivars and genetic clones of American blueberries. The novelty is the growth under Lithuanian climatic conditions. Chemometric tools, such as hierarchical cluster analysis and principal component analysis, indicate that the Woodman cultivar is different from other cranberry cultivars, as its samples contain twice the average total amounts of anthocyanins. A correlation was observed between the total anthocyanin content and the anti-radical and reducing activity of the in vitro extracts.

*Allium cepa* L. has a wide abundance worldwide, its versatility being a feature in culinary uses with the bulbs also showing many interesting medicinal uses, due to their high content of bioactive compounds. Barbero et al. [9] developed assisted extraction methods for the phenolic and anthocyanin compounds present, making use of a Box–Behnken design for their optimisation. Both methods show high repeatability and intermediate precision, short extraction times, and good recoveries.

Food by-products with high content of dietary fibre and free and bound bioactive compounds are usually discarded. Persimmon by-products are an interesting source of fibre and bioactive compounds, as demonstrated by Valero et al. [10]. The effects of solvent extraction of persimmon dietary fibre by-products after in vitro gastrointestinal digestion and probiotic bacterial fermentation on techno- and physico-functional properties were evaluated.

The purple potato variety is not well known although it is as rich in nutrients, amino acids, and starches as other potato varieties. In addition, it has a high anthocyanin content and its consumption is attractive in relation to human health. Barbero et al. [11] developed a methodology based on ultrasound-assisted extraction to achieve a higher anthocyanin yield. The method has been applied to successfully extract and quantify anthocyanins found in Vitelotte, Double Fun, Highland, and Violet Queen potatoes.

Native Chilean berries (rich in total polyphenols and anthocyanins) were studied by Alcudia et al. [12], and a large-scale extract of maqui berries was tested on intestinal epithelial and immune cells and shown to have potential as a nutraceutical agent with health benefits for the treatment of inflammatory bowel disease (IBD). Total polyphenol content (Folin–Cioculteau) and antioxidant capacity (DPPH, FRAP, and ORAC) were estimated, and the anthocyanin profile was assessed by ultra-high-performance liquid chromatography (UHPL-MS/MS).

Non-conventional extraction techniques meet the requirements of the food industry in terms of legal aspects, waste policy, safety, and environmental protection. However, the selection of a particular process is not an easy task and multiple factors are involved in planning the choice of the most suitable one. Tena et al. [13] provided an overview of recent applications in the field of anthocyanins extracted from different natural matrices, both by conventional and non-conventional techniques. Aspects such as the principles of the techniques involved, optimisation, technical progress, and industrial applications were considered and some useful recommendations were made.

The Achilles heel of anthocyanins is their lack of stability, which is affected by a number of factors such as pH, light, co-pigmentation, sulphites, ascorbic acid, oxygen, and enzymes. Diaconeasa et al. [14] reviewed all these factors affecting anthocyanin stability and degradation, assessing the impact of each parameter in order to minimise negative behaviour and consequently enhance the beneficial health effects.

The unstable nature of anthocyanins, which are affected as we have seen by changes in pH, oxidation, or high temperatures, requires the application of gentle non-thermal technologies for their extraction. Morata et al. [15] reviewed the characteristics, advantages, and disadvantages in the extraction of anthocyanins from grapes by applying non-thermal technologies such as Hugh hydrostatic pressure (HHP), ultra-high pressure homogenisation (UHPH), pulsed electric fields (PEF), ultrasound (US), irradiation, and pulsed light (PL). These techniques significantly increase extraction capacity while reducing extraction times and maintaining antioxidant capacity.

Chlorophylls and carotenoids are two families of antioxidants that include a large and complex number of compounds, present in daily food intake, with added value ingredients and functional properties. Their extraction and analysis require more powerful, precise, and accurate methods at hand, as well as a better understanding of the technical and biological context. Roca and Pérez-Gálvez [16] reviewed recent advances in the metabolomics of chlorophylls and carotenoids (pigmentomics), including material preparation and extraction procedures, and the use of instrumental techniques, e.g., spectroscopic and spectrometric (mass spectrometry to pigment metabolomics). The review also covered a critical account of studies showing the effects of biotic and abiotic stressors on living organisms, in which chlorophylls and carotenoids are involved.

Many thanks to the authors for their brilliant contributions, which have made this special issue possible. Many thanks also to Antioxidants for having us as guest editors for this special issue. Finally, we cannot leave out the reviewers for the important work they have done, which is worthy of mention and praise, and for which we also show our gratitude.
